# DNA polymerase preference determines PCR priming efficiency

**DOI:** 10.1186/1472-6750-14-10

**Published:** 2014-01-30

**Authors:** Wenjing Pan, Miranda Byrne-Steele, Chunlin Wang, Stanley Lu, Scott Clemmons, Robert J Zahorchak, Jian Han

**Affiliations:** 1 Biotechnology Science and Engineering Program, University of Alabama in Huntsville, Huntsville, AL 35899, USA; 2 HudsonAlpha Institute for Biotechnology, Huntsville, AL 35806, USA; 3 Stanford Genome Technology Center, Stanford University, Palo Alto, CA 94304, USA; 4 Diatherix Laboratories, Huntsville, AL 35806, USA

**Keywords:** PCR, DNA polymerase, Priming bias, Next generation sequencing, PPI, Polymerase preference index, iC-Architect

## Abstract

**Background:**

Polymerase chain reaction (PCR) is one of the most important developments in modern biotechnology. However, PCR is known to introduce biases, especially during multiplex reactions. Recent studies have implicated the DNA polymerase as the primary source of bias, particularly initiation of polymerization on the template strand. In our study, amplification from a synthetic library containing a 12 nucleotide random portion was used to provide an in-depth characterization of DNA polymerase priming bias. The synthetic library was amplified with three commercially available DNA polymerases using an anchored primer with a random 3’ hexamer end. After normalization, the next generation sequencing (NGS) results of the amplified libraries were directly compared to the unamplified synthetic library.

**Results:**

Here, high throughput sequencing was used to systematically demonstrate and characterize DNA polymerase priming bias. We demonstrate that certain sequence motifs are preferred over others as primers where the six nucleotide sequences at the 3’ end of the primer, as well as the sequences four base pairs downstream of the priming site, may influence priming efficiencies. DNA polymerases in the same family from two different commercial vendors prefer similar motifs, while another commercially available enzyme from a different DNA polymerase family prefers different motifs. Furthermore, the preferred priming motifs are GC-rich. The DNA polymerase preference for certain sequence motifs was verified by amplification from single-primer templates. We incorporated the observed DNA polymerase preference into a primer-design program that guides the placement of the primer to an optimal location on the template.

**Conclusions:**

DNA polymerase priming bias was characterized using a synthetic library amplification system and NGS. The characterization of DNA polymerase priming bias was then utilized to guide the primer-design process and demonstrate varying amplification efficiencies among three commercially available DNA polymerases. The results suggest that the interaction of the DNA polymerase with the primer:template junction during the initiation of DNA polymerization is very important in terms of overall amplification bias and has broader implications for both the primer design process and multiplex PCR.

## Background

The polymerase chain reaction (PCR) is one of the most important developments in modern biotechnology. However, PCR is known to introduce biases during amplification, particularly during multiplex PCR when several templates are amplified simultaneously [1,2]. Extreme base compositions (sequences with mostly G/C or A/T composition) are recognized to be problematic for both traditional Sanger sequencing and next generation sequencing platforms [3]. However, recent evaluations of biases generated in high throughput sequencing data have pinpointed the amplification step as the primary cause [4,5]. Factors such as thermocycler make, model, and ramping speed were demonstrated to affect the uniformity of the amplified library [4]. However, the DNA polymerase was identified as the primary source of bias with a variety of commercially available DNA polymerases skewing the amplification profile of the Neandertal genome with regard to GC content and template length [5].

While most applications focus on or even require the removal of the polymerase-dependent bias, we have taken an alternative approach in which we systematically define the amplification bias and utilize it to improve PCR success rates. The initiation of DNA polymerization on the template strand is a critical step in the polymerization process and is likely affected by differences among DNA polymerases and their interaction with the primer:template junction. In support of this notion, a study by Hansen et al. demonstrates that the use of random hexamer priming induces biases in the nucleotide composition at the beginning of transcriptome sequencing reads [6]. In our study, we used high throughput sequencing to test the hypothesis that DNA polymerases have a bias for different oligonucleotides used as primers to initiate DNA synthesis. In order to define this source of DNA polymerase bias, we carefully examined the contribution of the DNA sequence from a ten base pair (bp) window surrounding the primer:template junction including six base pairs (bps) of the primer:template duplex, which rests in the palm of the polymerase prior to nucleotide addition [7,8], and the four bps of single-stranded DNA template immediately following the 90° kink at the junction, which we termed the “runway”.

First, we created a synthetic sequencing library containing a twelve nucleotide random insertion (12 N) and flanking sequences without the use of amplification. This synthetic library (termed SL) provides a library of random sequences that reflect the complete pool of template sequences available prior to amplification. The SL was utilized as the template for several amplification experiments. The sequencing results of the amplification experiments were compared to the SL, which was sequenced directly (no amplification). After identifying sequence motifs that were preferentially amplified, single-primer templates were created in order to verify the DNA polymerase bias. We then developed a primer-design program, iC-Architect, which uses the observed bias in order to improve primer design.

## Methods

### Synthetic library and barcode production

**Figure 1:**
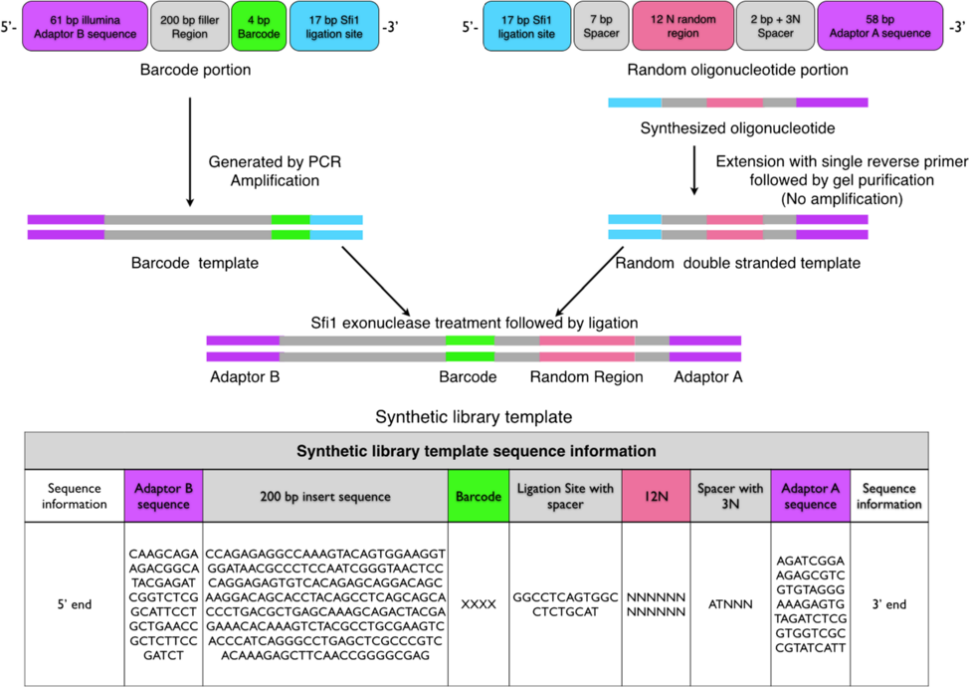
**Design of the barcode and synthetic random oligonucleotide used to create the SL.** The barcode portion was created through PCR amplification, while the synthesized random oligonucleotide was purchased and converted to a double stranded template. Both portions were digested with SfiI endonuclease, ligated, gel purified, and sequenced to produce the SL. Exact sequences used to generate the final synthetic library are provided.

The synthetic library (SL) was produced by the ligation of a barcode segment and a synthesized oligonucleotide (Figure 1). The synthesized oligonucleotide contains a 12 N random region, which was converted to a double stranded template as described below. The barcode portion of the SL design allows a unique barcode to be ligated to each of the different sample amplification test sets so that they can be pooled for high throughput sequencing. The barcode oligo consists of the Illumina adaptor B sequence, a filler region (200 bp long), and a 4 bp barcode followed by a SfiI site that matches the synthesized oligonucleotide’s 5’ end. The barcode portion was created by PCR amplification using a 200 bp portion of the human IgG C-kappa domain as the template to serve as the filler sequence. The filler sequence is used to make the end-product length optimal for bridge PCR during high throughput sequencing. Forward and reverse primers were designed so that they included the Illumina adaptor B sequence and the barcode (XXXX) with the SfiI ligation site, respectively. The forward primer utilized is as follows: 5’-CAAGCAGAAGACGGCATACGAGATCGGTCTCGGCATTCCTGCTGAACCGCTCTTCCGATCTCCAGAGAGGCCAAAGTACAG-3’, while the reverse primer is 5’-CGTAGGCCACTGAGGCCXXXXTCGCCCCGGTTGAAGCT. Each unique barcode was produced by amplification with Qiagen’s TopTaq Master Mix Kit in a Bio-Rad C1000 thermocycler as follows: initial denaturation at 94°C for 3 minutes; 35 cycles at 94°C for 30 s, 65°C for 30 s, and 72°C for 40 s; a final extension at 72°C for 10 minutes and then hold at 4°C. All amplified products were run on a 1.5% agarose gel, and the gel band of correct size was extracted and purified using Qiagen’s Qiaquick Gel Extraction Kit as per the manufacturer’s instructions.

The 99 bp synthesized oligo was purchased from IDT with standard desalting. The design includes a 17 bp SfiI ligation site, a 7 bp spacer, a 12 N random site, an additional 2 bp spacer followed by a 3 N random site to facilitate sequencing, and the Illumina sequencing adaptor A (Figure 1). Both the 7 bp and 2 bp spacer regions adjacent to the 12 N random site were required so that non-specific cleavage of the 12 N region did not occur during digestion with SfiI. The additional 3 N random site after the 2 bp spacer is required during sequencing so that the different clusters can be distinguished during first few cycles of sequencing. The synthesized oligo was converted to a double stranded template using a single reverse primer (5’-AATGATACGGCGACCACCGAGATCT-3’) and 35 cycles of 2 steps of annealing and extension including 55°C for 30 s and 72°C for 30 s with Qiagen’s HotStar HiFidelity DNA polymerase. An excess of cycles was used during this step to ensure that all of the single-stranded oligo was converted to double stranded template. The newly generated double stranded synthetic templates were gel purified, and the product was then directly ligated to the barcode portion after SfiI digestion. After ligation, the SL was gel purified after extraction from a 3% agarose gel and sequenced on the Illumina Hiseq 2000 platform using single end reads from adaptor A through SeqWright’s sequencing service. For all subsequent amplification experiments, a unique barcode portion was ligated to the amplified product, gel purified, and sequenced as described. The barcodes associated with a given amplification experiment are summarized in Additional file 1: Table S1.

### Amplification experiments

For all amplification experiments, 2 μL of SL at 50 ng/μL served as the template for amplification, and the same reverse primer as the SL production was used for all amplification experiments. All PCRs were performed as a 50 μL total reaction with 2 μL each of forward and reverse primer at 10 pmol/μL. All reactions were performed in the buffer system supplied by the respective DNA polymerase vendor without modification. For the amplified background experiment, the forward primer (5’-GCATGGCCTCAGTGGCCTCT-3’) was placed 4 bp upstream of the 12 N random site. Amplification was performed in a Bio-Rad C1000 thermocycler as follows: initial denaturation at 94°C for 3 minutes; 35 cycles at 94°C for 30 s, 65°C for 30 s, and 72°C for 30 s; a final extension at 72°C for 10 minutes and with a hold at 4°C.

For both the Promega and Qiagen’s Taq DNA polymerases, two repeat amplification experiments were performed for both types of polymerase, while one amplification experiment was performed with Qiagen’s HotStar HiFidelity DNA Polymerase. The analysis for only one commercial family B DNA polymerase was performed because we needed to ensure that we allotted enough sequencing reads for sufficient coverage of the 12 N random region of all of the currently described amplification experiments in one Illumina HiSeq sequencing lane. Since the synthetic library is considered a low diversity library, we expected the number of sequencing reads output to be at the lower end of the spectrum. Since we needed to include the different annealing temperatures and repeat experiments for the two Taq DNA polymerases, we limited the number of commercial DNA polymerases in the experiment to three. All polymerases utilized the same forward primer (5’-GCATGGCCTCAGTGGCCTCTGCATNNNNNN-3’) covering 6 nucleotides of the 12 N random portion of the template. All polymerases were subjected to the same cycling conditions, and amplification was performed as follows: initial denaturation at 94°C for 150 s; 35 cycles at 94°C for 30 s, 70–60°C touchdown for 120 s, and 72°C for 30 s; a final extension at 72°C for 10 minutes and then hold at 4°C. For the experiment testing different annealing temperatures, all PCR parameters remained the same with the exception of the annealing temperatures: 63, 65, and 67°C for 100 s.

### Statistical analysis of the observed bias value

**Figure 2:**
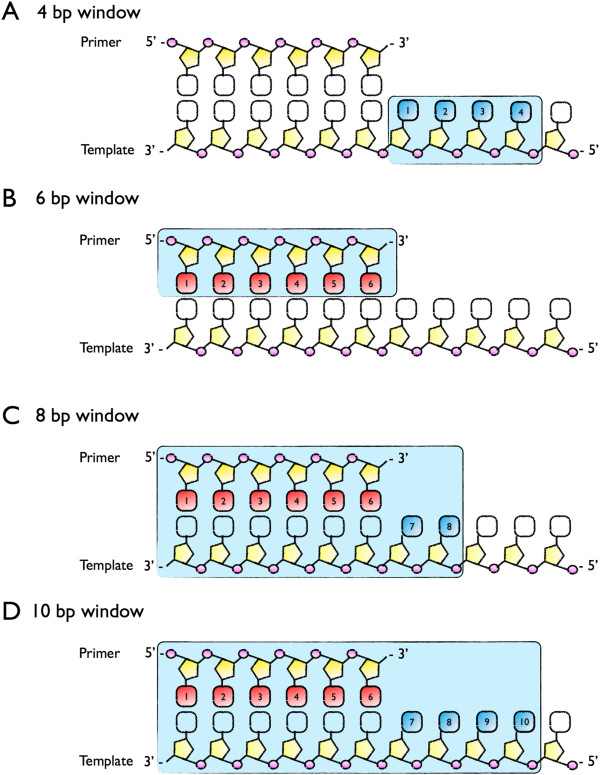
**Observation windows for the 12 N portion of the SL. (A)** The 4 bp window includes the nucleotides 4 bp ahead of the primer:template junction; **(B)** the 6 bp window includes the primer:template interaction; **(C)** the 8 bp window includes the primer:template interaction and 2 bp ahead of the primer:template junction; and **(D)** the 10 bp window includes the primer:template interaction and 4 bp ahead of the junction.

To assure that the proportion of each unique sequence in the SL was represented in the amplification experiments, we counted the reads for each sequence combination for four observation windows (4 bp-runway, 6 bp-primer:template interaction only, 8 bp- primer:template and runway, and 10 bp-primer:template and runway; Figure 2) for barcode 1 (the SL) and designated that result as the background for the generated template for all further analyses.

Since the number of the sequence reads differs between barcoded samples, normalization of the data was required prior to comparing samples. To do so, we adjusted the reads from data sets of differing scales to a notionally common scale. A z-score was calculated that adjusted a given sample size to that of the designated background sample (SL), and this standard score procedure was applied to all observation regions for all barcodes.

To ascertain the degree of bias in a given sample, a relative value for each specific sequence from the observation region was calculated. The normalized reads from the PCR amplified sample (NR_PCR_) were divided by the reads for the same sequence from the synthetic library (SR_SL_). For example, for a 6 bp observation window, assuming the specific DNA sequence “ATCGAT” results in 10 reads from the SL, and the normalized reads from the Taq polymerase PCR sample is 20, the “ATCGAT” at the end of primer has an observed bias value of 2. Theoretically, if there is no priming bias for the DNA polymerase, the observed bias value for all sequences in different observation regions should be similar to each other and close to a value of “1”. The observed bias value (OBV) formula follows:

$$\begin{array}{c} \mathrm{OBV}=\\ \; \end{array}\frac{{\mathrm{NR}}_{\mathrm{PCR}}}{{\mathrm{SR}}_{\mathrm{SL}}}\;$$

The statistics for all data comparisons are summarized in Additional file 2: Table S2.

### Single-primer test

Single-primer templates are templates with the same primer binding site on both the sense and anti-sense strands. In total, 24 unique single-primer templates were created and utilized for additional amplification experiments. The single-primer templates were produced by first designing primers that contained the primer site of interest and a portion of the human IgG C-kappa domain. Therefore, the forward primer consisted of a 19 nucleotide filler region, the 8 nucleotide primer site to be tested, and 19 nucleotides specific for the sense strand of the kappa domain, while the reverse primer included the identical filler and primer site with nucleotides specific for the anti-sense strand of the kappa domain (Additional file 3: Figure S1). The kappa domain was amplified as follows: initial denaturation at 94°C for 3 minutes; 35 cycles at 94°C for 30 s, 56°C for 30 s, and 72°C for 40 s; a final extension at 72°C for 10 minutes and then hold at 4°C. Twenty-four generated templates were separated on 1.5% agarose gel, and the band of the correct size was gel purified. All single-primer templates were adjusted to the same concentration of 0.001 ng/μL prior to the single-primer amplification test.

For each of the 24 templates, a unique single primer for each template was designed that includes the 19 nucleotide filler region and 6 nucleotides of the 8 nucleotide primer site. In each reaction, 2 μL of generated template and 2 μL of single primer at 10 pmol/μL were used with either Qiagen TopTaq or Qiagen HotStar HiFidelity DNA polymerase. End-point PCR was performed using two annealing temperatures, 57°C and 59°C for 30 s, with either 20 or 25 cycles. An internal control for each reaction was also performed with primers specific for an internal portion of the C-kappa region, which was the same for all 24 generated templates (forward-5’-TCTGTCTTCATCTTCCCGCCA-3’; reverse-5’AAGCTCTTTGTGACGGGCGAG-3’). Negative control amplification experiments, in which no template was added to the reaction, were performed, and no amplification was observed (data not shown).

## Results

### Amplification experiments and barcode analysis

**Figure 3:**
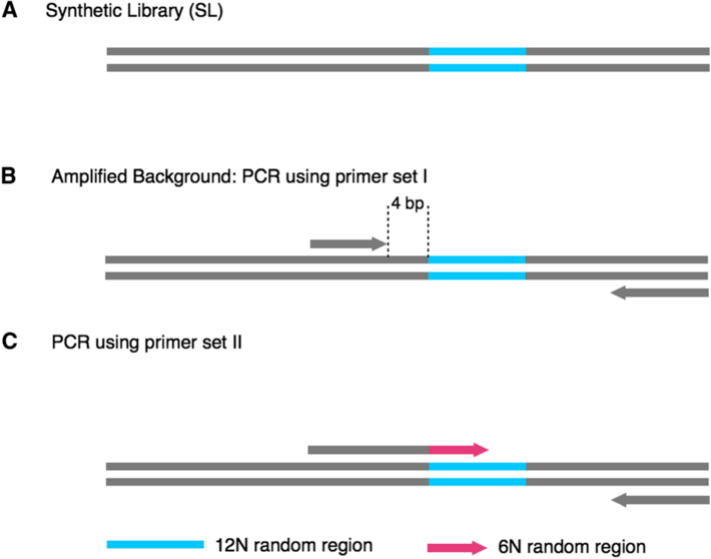
**Experimental set-up for the amplification of the SL with two different primer sets. (A)** The SL that contains a 12 N random region (blue) serves as the template for all additional amplification experiments; **(B)** in the amplified background experiment, the forward primer is placed 4 bp upstream of the 12 N region; **(C)** in the polymerase amplification tests, the forward primer (grey and pink) covers 6 N of the 12 N region.

In order to define the DNA polymerase bias, we created a synthetic library (SL) of sequences that are identical in sequence with the exception of a 12 nucleotide random insertion (Figure 3A). The SL was utilized as a template for a variety of amplification experiments, and all amplified libraries, including the non-amplified SL, were pooled and extensively sequenced using the Illumina HiSeq platform. Two primer sets were designed to amplify the SL. The forward and reverse primers of primer set I are located outside the random-insertion region (Figure 3B). The forward primer of primer set II is located at the boundary of the random-insertion region with six random nucleotides at the 3’ end of each primer (Figure 3C). The reverse primer of primer set II is identical to the reverse primer of primer set I. The SL was amplified with both primer sets I and II using three commercially available DNA polymerases: two family A DNA polymerases, Qiagen TopTaq (QTT-A) and Promega GoTaq (PGT-A); and one family B DNA polymerase Qiagen HotStar HighFidelity (QHH-B). Repeat amplification experiments were performed at several annealing temperatures (touchdown 70–60°C, 63°C, 65°C, and 67°C) as described in the Methods.

Prior to data analysis, all high throughput sequencing data were first separated into individual data sets by utilizing the barcodes associated with each experiment, and the data were normalized as described in the Methods. High throughput sequencing of the data sets returned 91,434,220 sequence reads, after filtering by the barcode sequences. The barcode sequences, number of associated reads, and the experimental description matching each barcode are shown in Additional file 1: Table S1. Sequencing of the unamplified SL confirmed that all of the possible combinations of template random nucleotide sequences are represented.

In order to analyze the data relevant to the random region, we filtered the data based on a 4 bp window (runway), 6 bp window (primer:template interaction), 8 bp window (primer:template interaction and 2 bps of runway), and 10 bp window (primer:template interaction and 4 bps of runway) as demonstrated in Figure 2. An observed bias value (OBV) was calculated and assigned to each sequence motif in the respective observation window (Methods). Since the ten bp observation window lacks sufficient read coverage (Additional file 1: Table S1), it was not utilized for further analysis due to lack of statistical significance.

### Observation of bias

**Figure 4:**
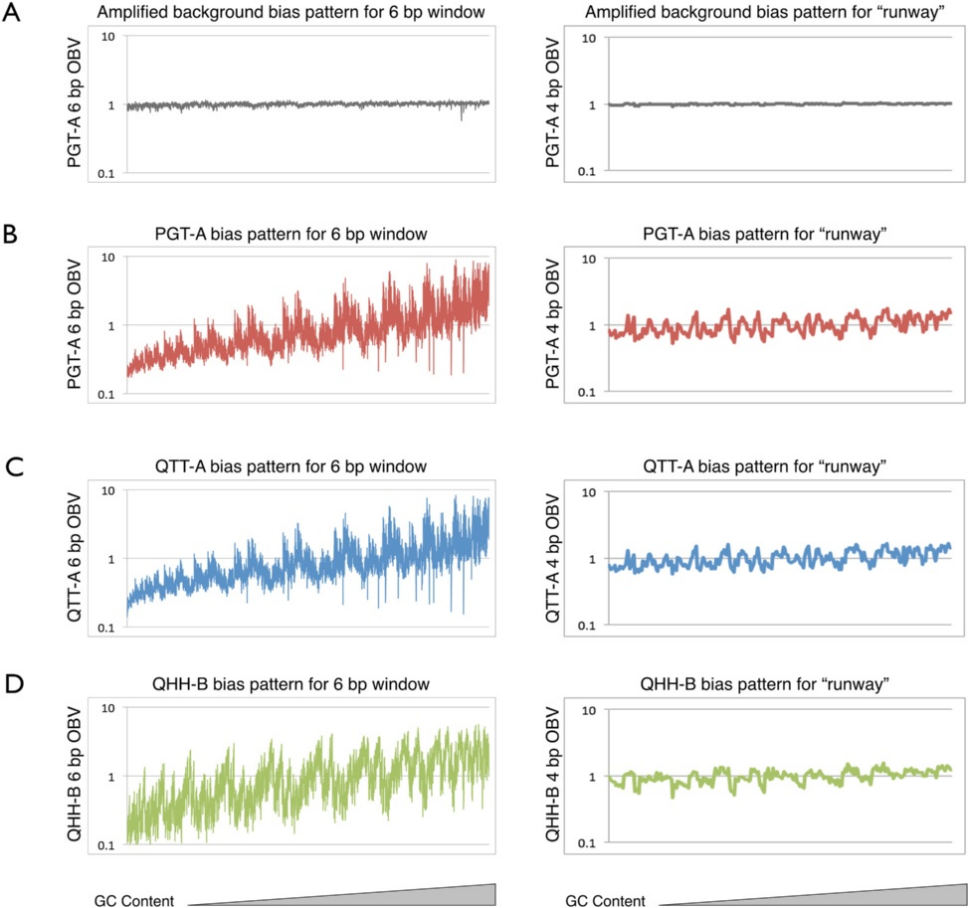
**Bias profiles for the SL and amplification experiments.** All bias profiles were created by plotting the observed bias value (OBV) for the 4 bp (n = 256) and 6 bp (n = 4,096) windows of observation on a logarithmic scale versus the amplified sequences, which were arranged from low to high GC content in the order of ATCG; **(A)** the bias observed after amplification with primer set I; **(B)** after amplification using primer set II with PGT-A DNA polymerase; **(C)** using primer set II with QTT-A DNA polymerase; **(D)** using primer set II with QHH-B DNA polymerase.

**Figure 5:**
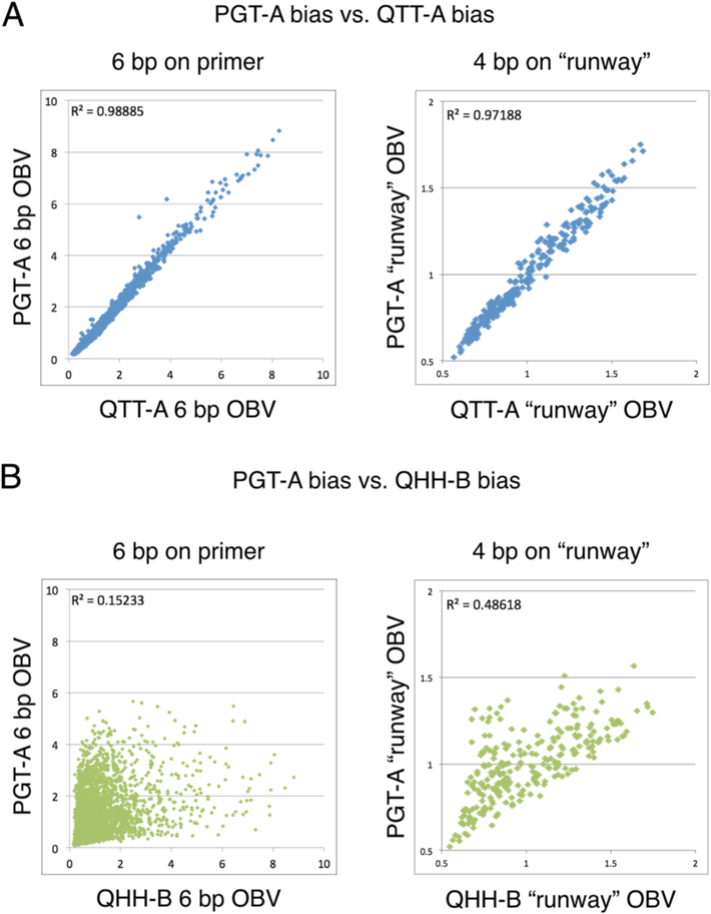
**Correlation of amplified libraries. (A)** A plot of the OBV of two family A polymerases (PGT-A versus QTT-A) demonstrates a high degree of correlation throughout the primer:template (6 bp) and runway (4 bp) regions; **(B)** a plot of the OBV of PGT-A versus OBV of QHH-B indicates that resulting amplification profile is weakly correlated for both the primer:template interaction (6 bp) and the runway (4 bp) for two differing families of polymerases.

When primer set I is used to amplify the SL (Figure 3B), the random sequences are faithfully amplified (Figure 4A). However, when a primer with six random bases (6 N) at the 3’ end is used to amplify the library (Figure 3C), priming bias is observed (Figure 4B-D). Furthermore, the observed bias is polymerase specific with the two commercially available family A DNA polymerases (QTT-A and PGT-A) preferring a set of sequence motifs different from those preferred by the family B DNA polymerase (QHH-B; Figure 5A-B). For instance, the sequence motif “GGGGGCGG” is the top-ranked one among 65,536 possible motifs for all the QTT-A DNA polymerase amplification experiments, including the touchdown, 63°C, 65°C, and 67°C annealing temperatures (Table 1). The same sequence motif is ranked second and third, respectively, for PGT-A polymerase. In contrast, this sequence motif is ranked 4,180 for the touchdown experiment of QHH-B. When comparing the top 30 ranked sequence motifs for the QTT-A touchdown PCR experiment across all family A DNA polymerase amplification experiments (including PGT-A), the same 30 sequence motifs appear within the top 87 of 65,536 possibilities. In contrast for the QHH-B touchdown experiment, these 30 sequence motifs are ranked from a range of 220 to 29,598 of 65,536 sequences (Table 1).

**Table 1:** A comparison of the top thirty ranked QTT-A sequences across all amplification experiments

**8 bp window sequence**	**Top 30 ranked QTT-A sequences: comparison of rank in other amplification experiments**							
	**QTT-A**	**QTT-AR**	**PGT-A**	**PGT-AR**	**QTT-A63**	**QTT-A65**	**QTT-A67**	**QHH-B**
**GGGGGC GG**	1	3	2	3	1	1	1	4180
**TTGGGC GG**	2	1	3	9	8	5	2	20382
**GGGCCG GG**	3	12	5	2	5	3	3	606
**TGGCCG GG**	4	5	4	4	6	6	6	3065
**GGTCCG GG**	5	6	1	1	3	2	4	1666
**GGGTGC GG**	6	9	6	13	16	12	9	2377
**GTGGGC GG**	7	11	10	5	9	7	11	1464
**TTGCCG GG**	8	8	8	7	22	21	20	13440
**GTGCCG GG**	9	13	7	8	7	13	17	220
**GGGGGC CG**	10	19	21	26	2	4	5	3950
**TGGTGC GG**	11	18	9	14	37	28	19	8530
**TTGGGC CG**	12	10	19	24	21	27	14	26404
**TGCCCG GG**	13	14	13	6	13	15	21	3370
**GGTCCG GC**	14	17	11	12	18	18	23	3802
**GGGGGC CA**	15	31	24	29	12	14	15	8559
**GGGCCG GC**	16	32	30	15	10	9	28	2255
**TGGCCG GC**	17	15	15	16	26	23	22	5312
**TTGTGC GG**	18	27	23	40	51	69	44	17564
**TGTCCG GG**	19	21	12	11	32	33	33	9598
**TTGGGC CA**	20	16	34	35	58	51	47	29598
**GGTGGC GG**	21	22	16	17	19	22	24	1890
**GGGGGC CC**	22	36	47	45	15	17	13	10961
**TTTGGC GG**	23	33	32	22	57	71	39	24345
**GGCCCG GG**	24	26	14	10	17	16	18	508
**TGGGCG GG**	25	29	29	23	29	47	40	5616
**GGGCGC GG**	26	87	33	38	25	34	42	1337
**TTGCCG GC**	27	23	31	32	46	52	65	18324
**TTGGGC GC**	28	24	51	57	92	62	60	25137
**GGGGGC GC**	29	47	58	64	27	24	27	9051
**GGGGGC TG**	30	35	55	80	4	8	10	8558

The top thirty sequences of the QTT-A touchdown PCR experiments were ranked and compared to the QTT-A repeat experiment (QTT-AR), the PGT-A and PGT-A repeat experiments (PGT-AR), the QTT-A experiments performed at 63°C, 65°C, and 67°C (QTT-A63, QTT-A65, and QTT-A67), and the QHH-B touchdown PCR amplification experiment.

There is positive amplification bias towards increasing GC content for the six base pairs of primer:template interaction for all of the commercial DNA polymerases tested. This is evident when the OBV for each sequence is plotted with the sequences arranged in order of increasing GC content (Figure 4B-D). For the top 1% of ranked sequences, the average GC content is 79% for the family A DNA polymerases and 83% for the QHH-B DNA polymerase. We also observed that the two bps located closest to the primer:template junction contained high GC content in sequences that were preferentially amplified. For instance, for the top 1% of ranked sequences of the 8 bp window, “GC” or “CG” is located at the 3’ end of the primer for 88-91% of the sequences across all of the family A DNA polymerase amplification experiments (Table 2). In contrast, 25% of the top-ranked sequences of QHH-B polymerase contain these sequence motifs at the 3’ end of the primer. The trend extends to the last three nucleotides on the 3’ end of the primer. The sequence motifs “CGC”, “CCG”, “GCG”, “GGC”, “TGC”, and “TCG” occur at the 3’ end of the primer for 87-90% of the top 1% of ranked sequences for the family A DNA polymerases and 22% for the family B DNA polymerase (Table 2). In contrast, QHH-B has a preference for “GC” or “CG” at the 5’ end of the 6 N portion of the primer with 89% of the top 1% of ranked sequences containing this motif, while only 6-12% of top ranked sequences from the family A experiments contain these sequences at their 5’ end. Therefore, the specific location of the GC content contributes to the amplification bias throughout the primer:template interaction. Interestingly, two GC-rich motifs at the 3’ end of the primer, “AGC” and “ACG”, had consistently poor amplification results across all polymerases with only 1% of top ranked QTT-A and PGT-A results and 4% of top ranked QHH-B sequences containing these motifs.

**Table 2:** The 8 bp window motif analyses for several DNA polymerase experiments

**Top 1% (655 in 65,536)**	**8 bp window motif analyses for several DNA polymerase experiments**							
	**QTT-A**	**QTT-AR**	**PGT-A**	**PGT-AR**	**QTT-A63**	**QTT-A65**	**QTT-A67**	**QHH-B**
**(GC,CG) motif at 3’ end of primer**	88%	89%	90%	90%	90%	91%	88%	25%
**(GC,CG) motif at 5’ end of 6 N portion of the primer**	8%	6%	8%	11%	12%	10%	8%	89%
**(TC) motif at first 2 bp of runway**	3%	4%	3%	3%	4%	4%	4%	19%
**(CC) motif at first 2 bp of runway**	28%	26%	28%	30%	23%	23%	25%	30%
**(CGC,CCG,GCG, GGC,TGC,TCG) motif at 3’ end of primer**	87%	88%	88%	89%	90%	90%	87%	22%
**(AGC,ACG) motif at 3’ end of primer**	1%	1%	1%	1%	1%	1%	1%	4%
**(ACG) motif at 3’ end of primer**	1%	1%	1%	1%	1%	1%	1%	2%
**(AGC) motif at 3’ end of primer**	0	0	0	0	0	0	0	2%

The motif percentage breakdown for the top 1% of ranked sequences are shown for several PCR experiments: QTT-A touchdown, QTT-AR, PGT-A, PGT-AR, QTT-A63, QTT-A65, QTT-A67, and the QHH-B touchdown PCR amplification experiment.

When the OBV for each runway motif is plotted with the sequences arranged in order of increasing GC content, the bias profile does not demonstrate a similar pattern to the primer:template interaction (Figure 4B-D). The sequence motif 3’-“TC”-5’ is preferred by QHH-B DNA polymerase in the first two positions after the primer:template junction (on the template sequence) with 19% of the top 1% of ranked sequences sharing this motif. In contrast, across all family A polymerase experiments, only 3-4% of top ranked sequences contain this motif at the beginning of the runway (Table 2). All of the DNA polymerases tested demonstrate a preference for 3’-“CC”-5’ in this position with 23-30% of family A and 30% of the family B top ranked sequences sharing this motif.

**Figure 6:**
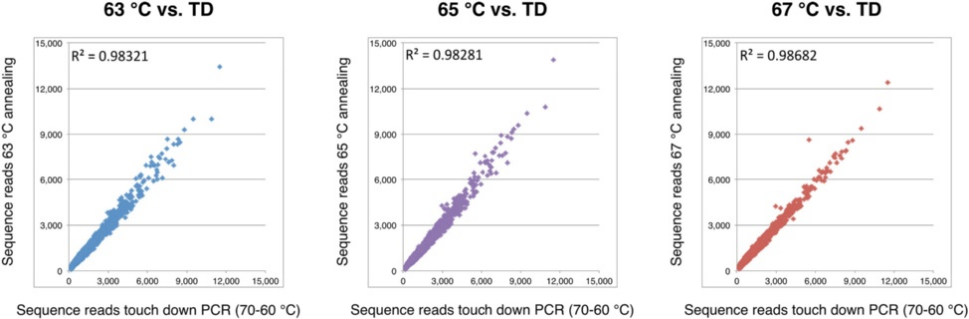
**Affect of annealing temperature on the amplified library.** Amplification was performed at 63, 65, and 67°C, and the sequenced reads were compared to touch down PCR experiments (70-60°C) for the 6 bp observation window.

In order to examine the effects of annealing temperature, we repeated amplification experiments at three additional annealing temperatures and compared the results to touchdown PCR experiments (Methods). All results were highly correlated with R^2^ > 0.98 (n = 4,096, p = 0.0000; Figure 6), indicating that the annealing temperature over the tested range did not have a significant effect on the bias profile for the six bp observation window.

Overall, QTT-A and PGT-A DNA polymerases have a similar amplification profile with an R^2^ of 0.98 (n = 4,096; p = 0.0000) for the primer:template interaction despite the fact that they were ordered from two separate vendors and likely work in different buffer conditions (Figure 5A). The similarity between the amplification profiles for both the primer:template interaction and the runway sequences of these two DNA polymerases is remarkable. However, we observed a different bias profile when the QTT-A polymerase was compared to the QHH-B DNA polymerase, a family B enzyme (Figure 5B). The bias profiles for the two different families of polymerases have an R^2^ of 0.15 (n = 4,096, p = 0.0000) for the primer:template interaction and an R^2^ of 0.49 (n = 4,096, p = 0.0000) for the runway sequences. An interaction between the primer and template DNA sequences independent of the polymerase cannot be the only factor leading to the observed bias; otherwise, all of the DNA polymerases would have amplified the SL similarly. The observed differences in amplification profiles are likely due to variation in the proprietary buffer conditions and structural differences between the two DNA polymerase families.

### Consideration of DNA polymerase bias during primer design

**Figure 7:**
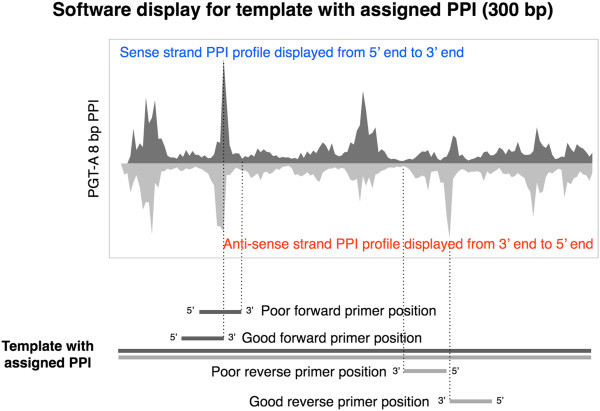
**Output of the iC-Architect software.** A graphical display of the output of the iC-Architect online software for a sample template strand. Maximum positions indicate the optimal placement for the 3’ end of the primer, while minimum positions should be avoided.

**Figure 8:**
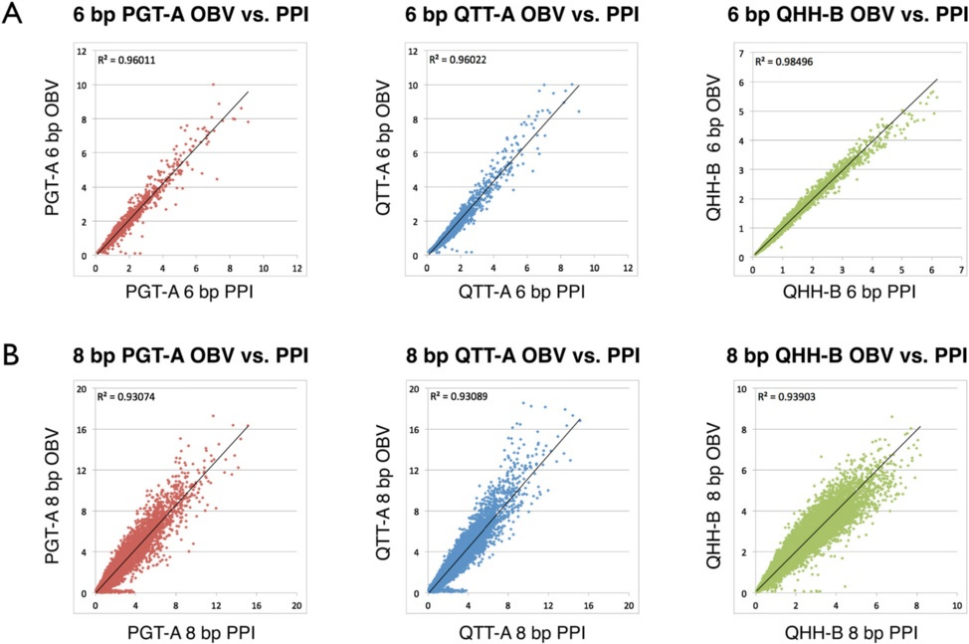
**Agreement between the modeled and observed bias.** The polymerase preference index (PPI) is highly correlated with the observed bias value (OBV) for both the **(A)** 6 bp and **(B)** 8 bp windows of observation for each of the DNA polymerase tested.

Based on the observed frequencies of DNA sequence motifs from the high throughput sequencing experiments, we developed a primer-design program called iC-Architect to aid PCR primer design by displaying the observed bias alongside target sequences (Additional file 4: Supplementary Methods). The software models the observed DNA polymerase preference by calculating an index value for a sliding eight base pair window, termed the polymerase preference index or PPI, and assigning the PPI value to the template strand (Additional file 5: Figure S2). An example of the iC-Architect display for a sample target sequence is provided in Figure 7. The software aids in positioning the 3’ end of the primer in places where the PPI value of the template is at a maximum. As demonstrated by the correlation coefficients in Figure 8A-B and Additional file 2: Table S2, the PPI accurately models the observed polymerase bias from high throughput sequencing experiments. However, there are a few notable outliers between the predicted and observed bias values. After examining the full-length sequence of these particular primers, significant hairpin secondary structure was predicted through IDT’s OligoAnalyzer Tool (http://www.idtdna.com/analyzer/Applications/OligoAnalyzer/), and poor amplification efficiency was observed during the amplification experiments. This underscores the importance of avoiding secondary structural elements such as hairpins when designing primers, and this additional step should be taken after considering the position of the primer on the template strand.

**Figure 9:**
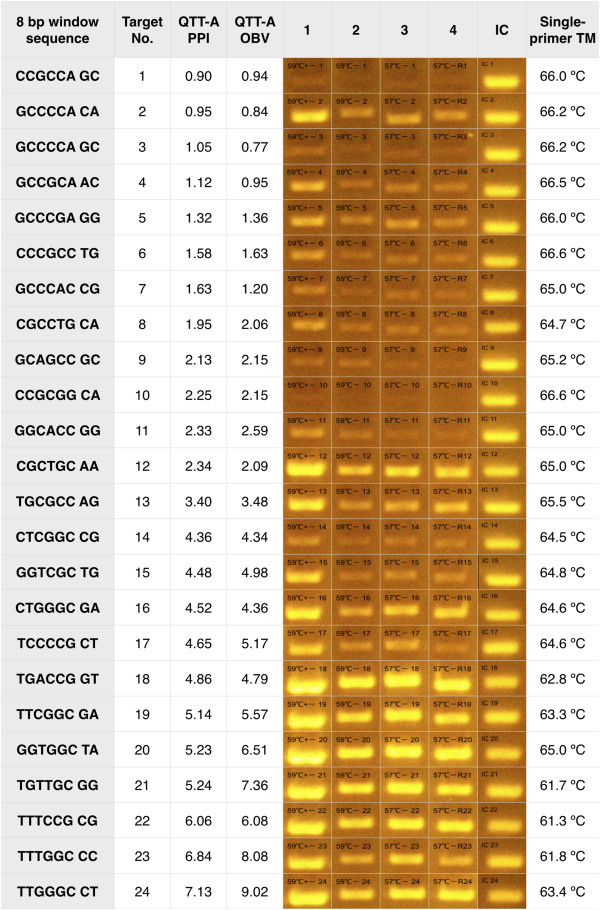
**Single primer experiments validating suggested primer-design locations from the iC-Architect software.** For each of the 24 single-primer templates, the agarose gel results are shown with calculated PPI and OBV values arranged from poor to good. Two repeat amplifications were performed at two temperatures: 59°C (lanes 1 and 2) and 57°C (lanes 3 and 4). All amplifications were stopped after 20 cycles, except lane 1, which was stopped at 25 cycles to demonstrate that 20 cycles was sufficient to see the difference in amplification. An internal control amplification was also performed on a portion of the sequence consistent with all templates. T_m_ values for the single-primers were calculated using the IDT OligoAnalyzer Tool.

We tested 24 primers that were predicted to have a range of polymerase preference from poor to good as indicated by the directly observed bias (Methods). In all cases, the observed bias was in agreement with the optimal primer position provided by the iC-Architect software and therefore further served to validate our software. Since we wanted the amplification to be attributed to only one primer at a time, we generated single-primer templates. These templates include the same single-primer binding site on both the forward and reverse strands. We designed the single primer to examine an eight bp window, which includes six bps of the primer:template and two nucleotides of the runway. The amplification profiles demonstrate that the bias both observed and predicted through the software can be replicated experimentally (Figure 9).

**Figure 10:**
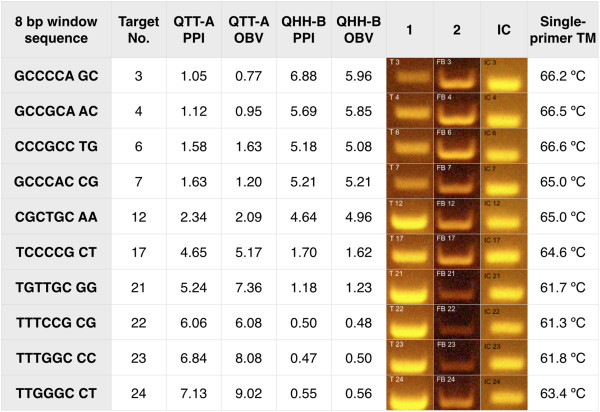
**Single primer experiments demonstrating the difference in amplification bias between different polymerase families.** For each of the 24 single-primer templates, a subset of sequences for the QHH-B DNA polymerase that have a PPI of the opposite trend with QTT-A DNA polymerase were selected. For agarose gel results, lane 1 shows the QTT-A results while lane 2 shows the QHH-B results, which has PPI values in reverse order. An internal control amplification was also performed on a portion of the sequence consistent with all templates.

We also selected a subset of primer sequences that showed positive amplification bias with the family A DNA polymerases and negative bias with the family B DNA polymerase, and vice versa. We performed the single-primer amplification tests with these two opposing subsets and were able to demonstrate that the two families of polymerases preferentially amplify certain sequences, yet fail to amplify others (Figure 10). Even though the two families of DNA polymerases have different bias profiles, the iC-Architect software is capable of predicting the best locations for the primer as long as the underlying data used to calculate the index refers to the specific commercially available DNA polymerase utilized in the experiment.

**Figure 11:**
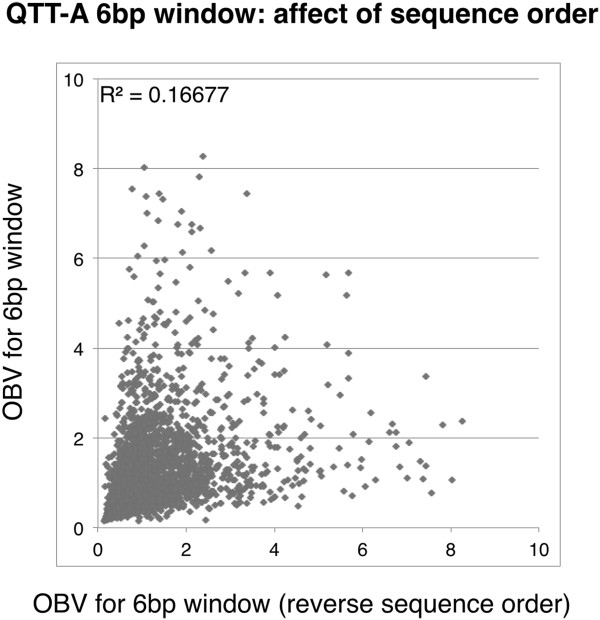
**Importance of the 3’ end of the primer sequence on amplification bias.** The OBV for the 6 bp window of (n = 4,096) was plotted against the OBV of the sequence of the reverse trimer order. For instance the OBV for the 6-mer “CCG AGG” is plotted against the OBV of “AGG CCG”. Although the sequences have the same GC content, the amplification results are not correlated.

The preferential amplification of certain sequences based on the type of DNA polymerase utilized indicates that GC and T_m_ calculations should not be the only considerations when designing primers. For example, in the single-primer template amplification experiments, the same single primer was used with two different types of DNA polymerases to amplify an identical template under the same reaction conditions. Although the primers have the same T_m_ and GC content, differences in amplification efficiency were readily evident (Figure 10). The differences in amplification efficiency may be related to both polymerase type and varying buffer conditions among commercial vendors. In other words, we cannot rule out that differing buffer conditions between the family A and family B DNA polymerase contribute to the observed difference or if it is actual structural differences between the two families of DNA polymerases (or a combination of both). Furthermore, the arrangement of nucleotides in the primer itself has a large impact on PCR success. There are many examples from the current high throughput sequencing experiment of sequences that have the same nucleotide content (and therefore, the same GC content), but vastly different outcomes in terms of amplification success due to the actual arrangement of the nucleotides in the sequence (Figure 11 and Additional file 6: Table S3). For example, the sequence CCG TTT has an OBV of 0.48, while the sequence TTT CCG has an OBV of 4.55. This is not to say that T_m_ and GC content should be ignored during primer design. They should be considered in addition to the DNA polymerase preference for specific sequence motifs.

## Discussion

Through the use of the next generation sequencing technology, the current study evaluated the polymerase preference by directly observing the priming efficiency of all possible hexamer primers. The crystal structure of catalytically active *Bacillus stearothermophilus* DNA polymerase crystals indicates that approximately ten bps of primer:template duplex DNA and four nucleotides of template in front of the primer template junction occupy the DNA binding site of the polymerase during synthesis [7,9]. In addition, the structure reveals a transition from A to B form DNA in the six bps adjacent to the primer:template junction. The tendency of a given nucleotide sequence toward A or B conformation is a key factor in many protein-DNA interactions [10,11]. For instance, when cyclic AMP receptor protein binds, the DNA undergoes a B to A like transition, and the binding is stronger when the central part of the DNA is in the A form [12]. Analogous to DNA polymerases, the RNA-DNA heteroduplex transiently formed during transcription [13] and the DNA in the active centers of HIV-1 reverse transcriptase [14] are also in the A form. It has been suggested that the stabilization of the A form may be critical for increasing the fidelity of DNA and RNA synthesis [15]. In this study, we observed that the two base pairs located at the primer:template junction contained a high GC content in sequences that were preferentially amplified. Several authors have demonstrated that poly (dG)-poly (dC) undergoes the transition from B form to A from more easily than poly (dA)-poly (dT), which tends to resist the B to A transition [12,16]. Indeed, the Gibb’s free energy for the B to A transition for GC rich trimeric motifs is lower than the transition of similar AT rich sequence motifs [16]. It is reasonable to consider that a primer:template interaction that has a tendency to be of the A form or a primer:template interaction that can be more easily conformed to the A form is preferentially bound by the DNA polymerase, ultimately resulting in biased amplification.

The purpose of the current study was to demonstrate and define DNA polymerase-dependent priming bias. Positive amplification bias towards increasing GC content was observed for the six base pairs of primer:template interaction for all of the commercial DNA polymerases tested, and several preferentially amplified sequence motifs were identified. Interestingly, Dabney et al. found that some commonly used polymerases strongly bias against amplification of endogenous DNA in favor of GC-rich microbial contamination [5]. In their study, Phusion HF and AmpliTaq Gold showed a very pronounced bias towards molecules with >50% GC. Another study by Hansen et al. demonstrated that random hexamer priming during the generation of cDNA induces biases in the beginning of nucleotide sequencing reads. In their study, they attempted to discern whether the high throughput sequencing reads originated from the sense strand by second-strand synthesis (from the DNA polymerase) or the antisense strand by first-strand synthesis (from the reverse transcriptase) [6]. Since both strands displayed a similar bias pattern, they concluded that the second strand DNA synthesis is likely being primed by remaining random hexamers in the solution. They also noted slight differences in the patterns in the sense and antisense strands which they attributed to different sequence specificities of the reverse transcriptase and DNA polymerase or the effect of nick priming [6]. Our data is in agreement with their conclusion and suggests that a majority of the bias is likely due to priming by the random hexamers and the preference of the DNA polymerase for certain primer:template junctions. The reverse transcriptase may also have preferential sequence motifs as the RNA-DNA heteroduplex in the active center of HIV-1 reverse transcriptase [14] is of the A form; however, reverse transcriptase bias was not addressed in the currently described experiments.

In our study, the four base pairs of single-stranded DNA template immediately following the junction, which we termed the “runway”, were also of interest because the polymerase can directly interact with the exposed bases of the single stranded template. This interaction can affect amplification. For instance, in archaeal family B DNA polymerases, the DNA polymerase possesses a read-ahead function in which polymerization will stall if an uracil is encountered 4 base pairs ahead of the primer:template junction [17,18]. This is not observed with family A DNA polymerases. The runway sequences in the described experiments did not have a clearly defined trend with regards to GC content like the primer:template interaction. However, some degree of bias was evident, and we were able to identify sequence motifs that were preferentially amplified. This suggests that when amplifying targets with universal primers, as in the case of certain multiplex PCR reactions, care should be taken to make sure that the primer-template junction is similar for all targets. If the primer:template junction varies ahead of the junction, this may result in the DNA polymerase preferentially amplifying “favorable” primer:template junctions over others.

In this work, all experiments were performed using the manufacturers’ polymerase-buffer systems directly. Modifying buffer conditions may influence enzyme and/or DNA configuration and achieve a successful amplification; however, this post-primer design effort may be avoided by adjusting the primer position slightly during the initial design phase. This is particularly useful when designing primers for multiplex reactions where inter-loci balancing is necessary to reduce conflicts among primers and targets. During the design of primers for multiplex reactions, the iC-Architect software can be used to select forward and reverse primers of relatively high and similar PPI values for multiple targets or to avoid regions with very low PPI values. Ultimately, the software identifies primers with preferred motifs on the 3’ end of the primer, which may be used to increase PCR success rates, especially when used in conjunction with T_m_ measurements.

## Conclusions

Random hexamer priming bias was analyzed by comparing the amplified products of a synthetic library to the unamplified synthetic library. Three commercially available DNA polymerases were utilized to amplify the library, and two of the DNA polymerases (both Taq) demonstrated remarkably similar amplification profiles, especially when compared to the third DNA polymerase of a different family. Preferentially amplified sequence motifs at the 3’ end of the primer were identified. These motifs demonstrated a marked GC-rich bias pattern. The identified bias patterns were used to guide primer design. Current primer design methods assume that all sequences can be used equally as priming sites, and melting temperature (T_m_) and GC measurements are the most important predictors of priming efficiency. However, our study demonstrates that the template sequence ahead of the primer:template junction and the 3’ end of the primer can have an effect on the amplification efficiency and should be considered in addition to T_m_ and GC measurements. In the future, we plan to extend the analysis to different commercially available DNA polymerases, particularly family B type DNA polymerases. In addition, it would be interesting to examine the effect of common PCR additives on the amplification bias profile and identify additives that may reduce bias. Use of the PPI software is available through the iC-Architect site at http://ic-architect.com/. After logging in, users can submit a template sequence. A downloadable csv file is generated, which provides the PPI profile for the template, and optimal primer positions are suggested. These results can be utilized together with T_m_ and GC calculations to guide the placement of a primer to preferential locations on the template.

## Abbreviations

PCR: Polymerase chain reaction; bp: base pair; bps: Base pairs; SL: Synthetic library; OBV: Observed bias value; QTT-A: Qiagen TopTaq Family A; PGT-A: Promega GoTaq Family A; QHH-B: Qiagen HotStar HighFidelity Family B; PPI: Polymerase preference index; IC: Internal control; NGS: Next generation sequencing; Tm: Melting temperature.

## Competing interests

Funding for project materials was provided by iCubate Inc. The authors declare financial competing interests for several of the authors listed. Jian Han is the chief scientific officer (CSO) and a shareholder for iCubate, Inc., while Stanley Lu and Scott Clemmons perform contract work for iCubate Inc. iCubate Inc. uses the iC-Architect software to design multiplex PCR panels for its open-platform diagnostic panels.

## Authors’ contributions

WP devised, executed all described experiments, performed data analysis, and contributed significantly to the writing of the manuscript. MBS aided with experimental design, data analysis and provided significant contribution to the writing of the manuscript. CW helped with data analysis and manuscript preparation. SL and SC developed the PPI software. RZ aided with experimental design and editing the manuscript. JH supervised the experimental design and execution and contributed to the writing of the manuscript. All authors read and approved the final manuscript.

## Supplementary Material

Additional file 1: Table S1 Barcodes and read coverage are presented. The barcode sequence, the number of total associated reads, the average number of reads per unique sequence in the 4 bp, 6 bp, 8 bp, and 10 bp windows, and the DNA polymerase used are provided for each of the pooled amplification experiments. During pooling, more of the SL and amplified background were included in the pool (as evidenced by the read distribution) to ensure that both of these libraries had an ample number of reads for downstream analysis. Click here for file

Additional file 2: Table S2 Statistical analysis of compared data sets. A statistical comparison of several data sets is provided with Pearson R, R^2^, p-value, and n. Click here for file

Additional file 3: Figure S1 Design for generating single-primer templates and their amplification. **(a)** The forward primer consists of a filler sequence, an 8 bp primer testing window and 18 nucleotides specific for a portion of the sense strand of the human IgG C-kappa domain. The reverse primer includes the same filler and 8 bp priming testing site, but includes 18 bp specific for the antisense strand of the kappa domain. After amplification, both the sense and antisense strand contain the same 8 bp sequence for single-primer testing. **(b)** After the templates are generated, gel purified, and the concentration normalized over all samples, a single primer, which serves as both forward and reverse, consisting of 19 bp of the filler region and 6 bp of the 8 bp priming site, is used in the amplification experiment. Click here for file

Additional file 4 Supplementary Methods. Click here for file

Additional file 5: Figure S2 An example demonstrating the PPI algorithm. First, the 8 bp window is divided into four sections: 1 dimeric scale (DiSc) and three trimeric scales (TrSc). The PPI value for each of the scales is based on the nucleotide sequence of the dimer or trimer and its relative position in the 8 bp window. The product of the DiSc and three TrScs is calculated and assigned to the 6th position of the 8 bp window. The entire 8 bp window is then slid 1 bp in the 3’ direction, and the process is repeated until the end of the template is reached. A PPI profile of both the sense and antisense strand can be generated in order to guide the placement of the 3’ end of the primer into a favorable position. Click here for file

Additional file 6: Table S3 The importance of the 3’ end of the primer sequence on amplification bias. A detailed demonstration of sequences that have the same GC content but very different amplification outcomes when amplified with QTT-A DNA polymerase. Click here for file
