# Supplementary material for: DNA polymerase preference determines PCR priming efficiency
Source: BMC Biotechnol. 2014 Jan 30;14:10. doi: 10.1186/1472-6750-14-10 (PMC3937175; doi:10.1186/1472-6750-14-10)
Supplement: Additional file 1: Table S1 — Barcodes and read coverage are presented. The barcode sequence, the number of total associated reads, the average number of reads per unique sequence in the 4 bp, 6 bp, 8 bp, and 10 bp windows, and the DNA polymerase used are provided for each of the pooled amplification experiments. During pooling, more of the SL and amplified background were included in the pool (as evidenced by the read distribution) to ensure that both of these libraries had an ample number of reads for downstream analysis. [file 1472-6750-14-10-S1.pdf]

Barcode Number	Barcode Sequence	Total Number of Reads	Average Number of Reads Per Unique Sequence in a Given Observation Window (4bp, 6bp, 8bp, 10bp)				Barcode and DNA Polymerase Description
			4 bp	6 bp	8 bp	10 bp	
1	GCAT	9,720,533	37,971	2,373	148	10	SL: template used for all PCR amplification experiments
2	CCAA	11,462,176	44,774	2,798	175	11	Amplified background: Primer placed 4 bp outside of the 12 N region
3	GATC	4,925,811	19,241	1,203	75	5	PGT-A pol 6N amplification product
4	GCTT	5,154,225	20,134	1,258	79	5	PGT-A pol 6N amplification product: repeat
5	GGCC	4,786,732	18,698	1,169	73	5	QTT-A pol 6N amplification product
6	ATCG	5,904,773	23,066	1,442	90	6	QTT-A pol 6N amplification product: repeat
7	CGGT	9,165,718	35,804	2,238	140	9	QHH-B pol 6N amplification product
8	GTCA	5,704,314	22,282	1,393	87	6	QTT-A pol 6N product (63 °C Annealing)
9	TTGG	5,014,614	19,588	1,224	77	5	QTT-A pol 6N product (65 °C Annealing)
10	CCGG	5,480,506	21,408	1,338	84	5	QTT-A pol 6N product (67 °C Annealing)

Pan et al, Additional file 1 – Table S1

Barcodes and read coverage are presented. The barcode sequence, the number of total associated reads, the average number of reads per unique sequence in the 4 bp, 6 bp, 8 bp, and 10 bp windows, and the DNA polymerase used are provided for each of the pooled amplification experiments. During pooling, more of the SL and amplified background were included in the pool (as evidenced by the read distribution) to ensure that both of these libraries had an ample number of reads for downstream analysis.
