# Supplementary material for: DNA polymerase preference determines PCR priming efficiency
Source: BMC Biotechnol. 2014 Jan 30;14:10. doi: 10.1186/1472-6750-14-10 (PMC3937175; doi:10.1186/1472-6750-14-10)
Supplement: Additional file 2: Table S2 — Statistical analysis of compared data sets. A statistical comparison of several data sets is provided with Pearson R, R2, p-value, and n. [file 1472-6750-14-10-S2.pdf]

4 bp “runway” relative read bias against SL	Pearson R	R^2	p =	N =
**Amplified Background**	0.994	0.988	0.000000	256
**Promega GoTaq**	0.484	0.234	0.000000	256
**Qiagen TopTaq**	0.462	0.213	0.000000	256
**Qiagen HotStar Hifidelity**	0.680	0.462	0.000000	256
**6 bp relative read bias against SL**				
**Amplified Background**	0.971	0.943	0.000000	4, 096
**Promega GoTaq**	0.161	0.026	0.000000	4, 096
**Qiagen TopTaq**	0.167	0.028	0.000000	4, 096
**Qiagen HotStar Hifidelity**	-0.220	0.048	0.000000	4, 096
**8 bp relative read bias Against SL**				
**Amplified Background**	0.904	0.817	0.000000	65,536
**Promega GoTaq**	0.165	0.027	0.000000	65,536
**Qiagen TopTaq**	0.159	0.025	0.000000	65,536
**Qiagen HotStar Hifidelity**	-0.102	0.010	0.000000	65,536
**Promega GoTaq vs. Qiagen TopTaq Bias**				
**4 bp “runway”**	0.986	0.972	0.000000	256
**6 bp**	0.994	0.988	0.000000	4,096
**8 bp**	0.974	0.949	0.000000	65,536
**Promega GoTaq vs. Qiagen HotStar HiFidelity Bias**				
**4 bp “runway”**	0.697	0.486	0.000000	256
**6 bp**	0.390	0.152	0.000000	4,096
**8 bp**	0.402	0.162	0.000000	65,536
**OBV vs. PPI**				
**6 bp Promega GoTaq**	0.980	0.960	0.000000	4, 096
**6 bp Qiagen TopTaq**	0.980	0.960	0.000000	4,096
**6 bp Qiagen HotStar HiFidelity**	0.992	0.984	0.000000	4,096
**8 bp Promega GoTaq**	0.965	0.931	0.000000	65,536
**8 bp Qiagen TopTaq**	0.965	0.931	0.000000	65,536
**8 bp Qiagen HotStar HiFidelity**	0.969	0.939	0.000000	65,536

Pan et al, Additional file 2 – Table S2

Statistical analysis of compared data sets. A statistical comparison of several data sets is provided with Pearson R, R^2, p-value, and n.
