# Supplementary material for: DNA polymerase preference determines PCR priming efficiency
Source: BMC Biotechnol. 2014 Jan 30;14:10. doi: 10.1186/1472-6750-14-10 (PMC3937175; doi:10.1186/1472-6750-14-10)
Supplement: Additional file 3: Figure S1 — Design for generating single-primer templates and their amplification. (a) The forward primer consists of a filler sequence, an 8 bp primer testing window and 18 nucleotides specific for a portion of the sense strand of the human IgG C-kappa domain. The reverse primer includes the same filler and 8 bp priming testing site, but includes 18 bp specific for the antisense strand of the kappa domain. After amplification, both the sense and antisense strand contain the same 8 bp sequence for single-primer testing. (b) After the templates are generated, gel purified, and the concentration normalized over all samples, a single primer, which serves as both forward and reverse, consisting of 19 bp of the filler region and 6 bp of the 8 bp priming site, is used in the amplification experiment. [file 1472-6750-14-10-S3.pdf]

a

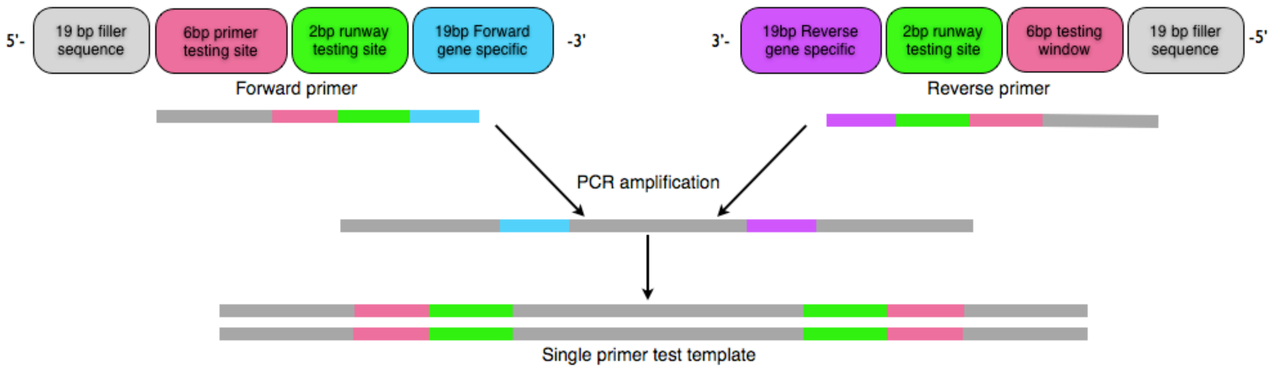

Single primer test template sequence information									
Sequence information	Forward filler	6bp priming site	2bp runway	300 bp insert sequence	2bp runway	6bp priming site	Reverse filler	Sequence information
5' end	GCCTCA GTGGCC TCTGCAT	XXXXXX (TTTGGC)	XX (CC)	AGAACCGTGGCTGCACCATCTGTCTTCATCTTCCCGCCATCTGA TGAGCAGTTGAAATCTGGAACTGCCTCTGTTGTGTGCCTGCTG AATAACTTCTATCCCAGAGAGGCCAAAGTACAGTGGAAGGTGGA TAACGCCCTCCAATCGGGTAACTCCCAGGAGAGTGTCACAGAG CAGGACAGCAAGGACAGCACCTACAGCCTCAGCAGCACCCTG ACGCTGAGCAAAGCAGACTACGAGAAACACAAAGTCTACGCCT GCGAAGTCACCCATCAGGGCCTGAGCTCGCCCGTCACAAAG	XX (GG)	XXXXXX (GCCAAA)	ATGCAGA GGCCAC TGAGGC	3' end

b

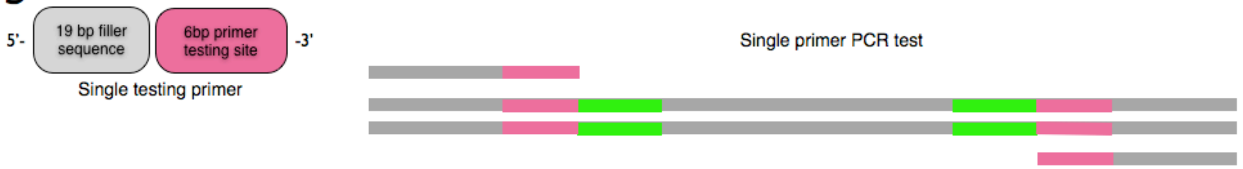

Pan et al, Additional file 3 – Figure S1

Design for generating single-primer templates and their amplification. (a) The forward primer consists of a filler sequence, an 8 bp primer testing window and 18 nucleotides specific for a portion of the sense strand of the human IgG C-kappa domain. The reverse primer includes the same filler and 8 bp priming testing site, but includes 18 bp specific for the antisense strand of the kappa domain. After amplification, both the sense and antisense strand contain the same 8 bp sequence for single-primer testing. (b) After the templates are generated, gel purified, and the concentration normalized over all samples, a single primer, which serves as both forward and reverse, consisting of 19 bp of the filler region and 6 bp of the 8 bp priming site, is used in the amplification experiment.
