# Supplementary material for: DNA polymerase preference determines PCR priming efficiency
Source: BMC Biotechnol. 2014 Jan 30;14:10. doi: 10.1186/1472-6750-14-10 (PMC3937175; doi:10.1186/1472-6750-14-10)
Supplement: Additional file 4 — Supplementary Methods. [file 1472-6750-14-10-S4.pdf]

Pan et al, Additional file 4 – Supplementary Methods

Polymerase Preference Index Algorithm

Previous studies have demonstrated that a trimeric scale (TrSc) describes the sequence-dependent DNA conformational propensities more accurately than a dimeric scale (16). The conformational transition of DNA from A to B form throughout six base pairs encompassing the primer:template junction may be an important factor in the ability of the DNA polymerase to initiate synthesis. Therefore, we decided to utilize one dimeric scale (DiSc) and three TrScs to model the observed polymerase bias for the eight bp observation window. To do so, we first noted by observing the frequencies of different trinucleotides in the context of an eight bp window, that the frequency distribution of a given trinucleotide sequence was independent of the rest of the sequence in the corresponding window. Since the trinucleotide sequences can be treated independently, we were able to assign an observed bias value (OBV) to the DiSc and each TrSc. The OBV comes directly from the high throughput sequencing experiments (**Methods**). The nucleotide scales divide the eight bp window into four sections. For each of the sections, the OBV for the DiSc and each of the TrSc is assigned based on the nucleotide sequence of the scale and its relative location in the eight bp window. The DiSc is always situated in a position 1-2 (the farthest position upstream of the primer:template junction). Once this process is completed for a given eight bp window, the OBV values for each of the four sections are multiplied in order to calculate the PPI value for the eight bp window (**Additional file 5: Figure S2**). Due to the shorter length of the six bp window, we used a slightly modified version of the algorithm to calculate the PPI in order to compare to the OBV for the six bp observation window. The PPI for a 6 bp region was calculated by using the product of the OBV for one dimeric scale and two trimeric scales.

When calculating the PPI for an entire template strand, the PPI for an eight bp window is calculated as discussed previously and assigned to the sixth nucleotide position of the eight bp window, which corresponds to the position for the last bp on the primer's 3' end. The entire eight bp window is then shifted one bp in the 3' direction, and the process is repeated for the next eight bp window until the end of the template reached. The process is completed for both the sense and the antisense strand independently in order to generate the PPI for both strands.
