# Supplementary material for: DNA polymerase preference determines PCR priming efficiency
Source: BMC Biotechnol. 2014 Jan 30;14:10. doi: 10.1186/1472-6750-14-10 (PMC3937175; doi:10.1186/1472-6750-14-10)
Supplement: Additional file 5: Figure S2 — An example demonstrating the PPI algorithm. First, the 8 bp window is divided into four sections: 1 dimeric scale (DiSc) and three trimeric scales (TrSc). The PPI value for each of the scales is based on the nucleotide sequence of the dimer or trimer and its relative position in the 8 bp window. The product of the DiSc and three TrScs is calculated and assigned to the 6th position of the 8 bp window. The entire 8 bp window is then slid 1 bp in the 3’ direction, and the process is repeated until the end of the template is reached. A PPI profile of both the sense and antisense strand can be generated in order to guide the placement of the 3’ end of the primer into a favorable position. [file 1472-6750-14-10-S5.pdf]

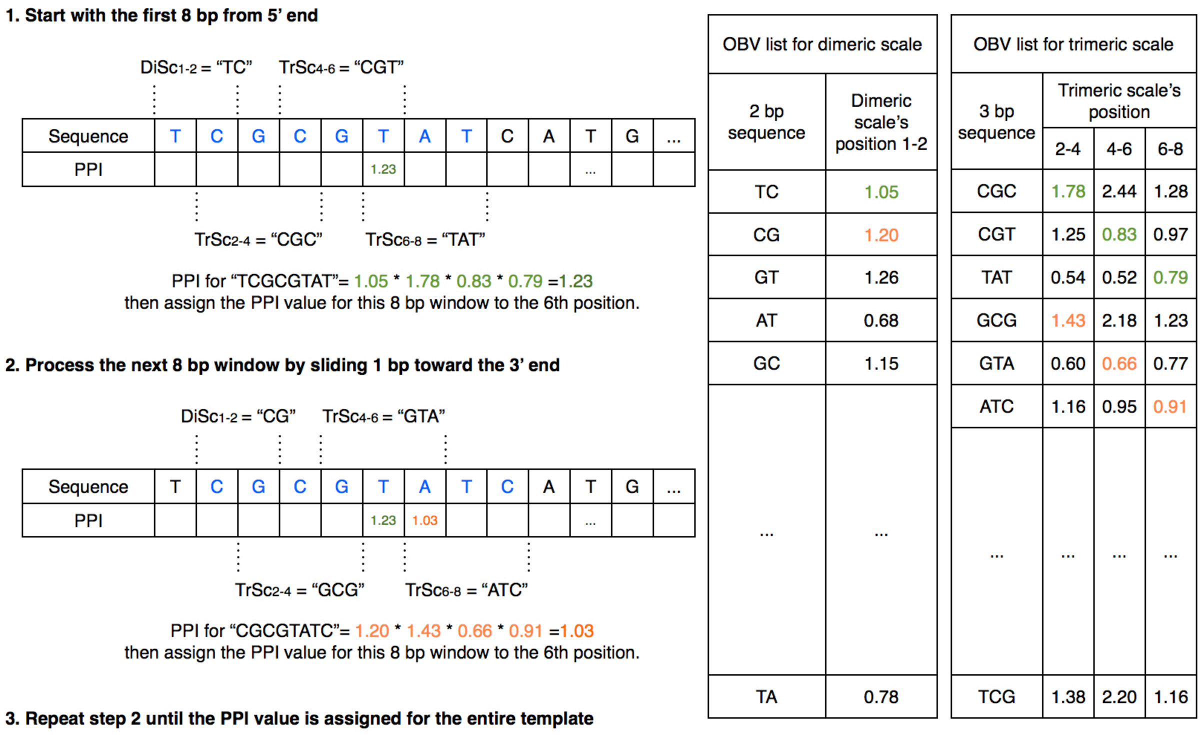

Pan et al, Additional file 5 – Figure S2

An example demonstrating the PPI algorithm. First, the 8 bp window is divided into four sections: 1 dimeric scale (DiSc) and three trimeric scales (TrSc). The PPI value for each of the scales is based on the nucleotide sequence of the dimer or trimer and its relative position in the 8 bp window. The product of the DiSc and three TrScs is calculated and assigned to the 6th position of the 8 bp window. The entire 8 bp window is then slid 1 bp in the 3’ direction, and the process is repeated until the end of the template is reached. A PPI profile of both the sense and antisense strand can be generated in order to guide the placement of the 3’ end of the primer into a favorable position.
