# Supplementary material for: DNA polymerase preference determines PCR priming efficiency
Source: BMC Biotechnol. 2014 Jan 30;14:10. doi: 10.1186/1472-6750-14-10 (PMC3937175; doi:10.1186/1472-6750-14-10)
Supplement: Additional file 6: Table S3 — The importance of the 3’ end of the primer sequence on amplification bias. A detailed demonstration of sequences that have the same GC content but very different amplification outcomes when amplified with QTT-A DNA polymerase. [file 1472-6750-14-10-S6.pdf]

NO.	GC (%)	6bp Sequence	6bp OBV	6bp Sequence (Reverse)	6bp OBV
1	50	CCG TTT	0.48	TTT CCG	4.55
2	50	GCC TTT	0.51	TTT GCC	3.15
3	83.3	CCG CAG	0.52	CAG CCG	3.16
4	66.7	CCG GAT	0.54	GAT CCG	3.37
5	66.7	CCG AGT	0.56	AGT CCG	3.09
6	50	CCG TTA	0.57	TTA CCG	3.22
7	50	CGC TTT	0.58	TTT CGC	3.76
8	66.7	CCG ATG	0.62	ATG CCG	3.92
9	33.3	TGC TTT	0.66	TTT TGC	3.19
10	83.3	CCG AGG	0.67	AGG CCG	3.99
11	83.3	CCG GAG	0.67	GAG CCG	4.24
12	66.7	CCG TCT	0.67	TCT CCG	3.45
13	66.7	CCG TAG	0.69	TAG CCG	4.00
14	66.7	CCG GTT	0.70	GTT CCG	4.63
15	66.7	GCC TTC	0.70	TTC GCC	3.22
16	50	GGC TTT	0.72	TTT GGC	5.76
17	50	GCG TTT	0.77	TTT GCG	3.48
18	66.7	CCG TTG	0.78	TTG CCG	7.56
19	66.7	CCG TGA	0.80	TGA CCG	3.90
20	66.7	CCG TTC	0.81	TTC CCG	5.59
21	66.7	CCG GTA	0.82	GTA CCG	3.19
22	66.7	CGC TAG	0.84	TAG CGC	3.26
23	66.7	GGC CTT	0.85	CTT GGC	3.30
24	66.7	GCC TGT	0.87	TGT GCC	4.14
25	66.7	CCG TAC	0.91	TAC CCG	3.70
26	66.7	CCG TGT	0.91	TGT CCG	6.06
27	83.3	CGC AGG	0.92	AGG CGC	3.75
28	83.3	CCG CTC	0.92	CTC CCG	3.37
29	83.3	CCC TGG	0.93	TGG CCC	3.58
30	66.7	GGC TCT	0.93	TCT GGC	3.73

Pan et al, Additional file 6 – Table S3

The importance of the 3’ end of the primer sequence on amplification bias. A detailed demonstration of sequences that have the same GC content but very different amplification outcomes when amplified with QTT-A DNA polymerase.
